# β-Hydroxybutyrate Oxidation Promotes the Accumulation of Immunometabolites in Activated Microglia Cells

**DOI:** 10.3390/metabo10090346

**Published:** 2020-08-26

**Authors:** Adrian Benito, Nabil Hajji, Kevin O’Neill, Hector C. Keun, Nelofer Syed

**Affiliations:** 1 Division of Neuroscience, Department of Brain Sciences, Imperial College London, London W12 0NN, UK; a.benito-mauricio@imperial.ac.uk (A.B.); n.hajji@imperial.ac.uk (N.H.); kevin.oneill@nhs.net (K.O.); 2 Division of Cancer, Department of Surgery and Cancer, Imperial College London, London W12 0NN, UK; 3 Division of Systems Medicine, Department of Metabolism, Digestion and Reproduction, Imperial College London, London W12 0NN, UK

**Keywords:** microglia, β-hydroxybutyrate, metabolic reprogramming, stable-isotope tracing, metabolomics

## Abstract

Metabolic regulation of immune cells has arisen as a critical set of processes required for appropriate response to immunological signals. While our knowledge in this area has rapidly expanded in leukocytes, much less is known about the metabolic regulation of brain-resident microglia. In particular, the role of alternative nutrients to glucose remains poorly understood. Here, we use stable-isotope (^13^C) tracing strategies and metabolomics to characterize the oxidative metabolism of β-hydroxybutyrate (BHB) in human (HMC3) and murine (BV2) microglia cells and the interplay with glucose in resting and LPS-activated BV2 cells. We found that BHB is imported and oxidised in the TCA cycle in both cell lines with a subsequent increase in the cytosolic NADH:NAD^+^ ratio. In BV2 cells, stimulation with LPS upregulated the glycolytic flux, increased the cytosolic NADH:NAD^+^ ratio and promoted the accumulation of the glycolytic intermediate dihydroxyacetone phosphate (DHAP). The addition of BHB enhanced LPS-induced accumulation of DHAP and promoted glucose-derived lactate export. BHB also synergistically increased LPS-induced accumulation of succinate and other key immunometabolites, such as α-ketoglutarate and fumarate generated by the TCA cycle. Finally, BHB upregulated the expression of a key pro-inflammatory (M1 polarisation) marker gene, *NOS2*, in BV2 cells activated with LPS. In conclusion, we identify BHB as a potentially immunomodulatory metabolic substrate for microglia that promotes metabolic reprogramming during pro-inflammatory response.

## 1. Introduction

Microglia are the resident immune cells of the central nervous system (CNS) and represent approximately 10% of CNS cells in a healthy brain and spinal cord. These cells have recently attracted renewed interest because of the critical role they play in major brain diseases, such as dementia [1], stroke [2], and brain tumours [3]. In brain disease or upon immune challenge, resting microglia adopt programmatic changes associated with the release of cytokines and chemokines. These polarised cells have been traditionally categorised as having either a pro-inflammatory (M1 type) or anti-inflammatory (M2 type) states depending on the expression of a few molecular markers. Molecules like lipopolysaccharide (LPS) and interferon-γ (IFN-γ) are strong promoters of M1 polarisation, whereas IL-4 induces M2 polarisation. However, accumulating evidence has demonstrated the limitations of the M1/M2 conceptual framework, and the polarisation of microglia remains a subject of intense scientific debate [4].

An increasing body of knowledge attributes a crucial role to cellular metabolism in the regulation of microglial function and polarisation in both health and disease [5,6,7,8]. The extracellular metabolic environment and the dynamic changes in the intracellular metabolic milieu orchestrated by metabolic reactions modulate the response to immune signals. In peripheral immune cells, the mechanisms by which inflammation affects energy metabolism are now well-established [9,10]. Similarly, in microglia, recent findings indicate that this immune cell type engages with different metabolic pathways depending on the pattern of stimulation [11,12]. However, much less is known about how energy metabolism and the metabolic microenvironment affects immune responses [13]. Observations in immunometabolism have reported that peripheral immune cells can adapt to fluctuating environmental challenges by metabolising alternative nutrients other than glucose, such as acetate [14], amino acids [15], or fatty acids [13,16]. In microglia, this phenomenon of the so-called metabolic flexibility and the utilisation of alternative substrates other that glucose is still poorly understood. Only very recently it has been reported that microglia can switch to utilise glutamine as an alternative fuel in the absence of glucose to support microglial function [17,18]. The matter of metabolic flexibility gains relevance as more evidence emerges regarding the significance of the metabolic microenvironment in regulating the immune function. Recently, the term immunometabolites or cytokine-like metabolites has been coined to describe the metabolites succinate, itaconate, lactate, fumarate, and α-ketoglutarate [19,20]. These are metabolites that have critical functions in leukocyte activation and differentiation that are independent of their conventional roles in biosynthesis and bioenergetics.

Dietary interventions have shown potential to alter the metabolic environment and subsequently fine-tune the immune system [21,22,23,24,25,26,27]. Amongst these, ketogenic diets have been one of the most popular, particularly in the treatment of diseases of the brain like epilepsy and glioma [28,29,30,31,32]. Amongst the plethora of effects associated with this diet, the increase in the endogenous production of the ketone body β-hydroxybutyrate (BHB) is one of the most biologically significant. Proof of the relevance of this metabolite is the fact that, when administered alone, BHB can recapitulate the beneficial effects of the ketogenic diet in some conditions [22,33,34]. BHB is a four-carbon molecule produced from hepatic fatty acid oxidation under conditions of energy restriction. It can act as a signalling molecule by direct binding to the G-protein-coupled receptor GPR109A or as a histone deacetylase (HDAC) inhibitor or indirectly, via oxidative metabolism with the subsequent production of acetyl-CoA and NADH [35]. Whereas the direct signalling effects of BHB in brain and microglia have been extensively studied in different systems [33,36,37,38,39,40,41], the metabolism of BHB in microglia has not been previously characterised. This lack of knowledge represents a barrier towards our full understanding of the effects of this nutrient in microglia and in the wider context of brain-related diseases. Hence, given the central role of metabolic signalling and reprogramming in immunity, understanding the metabolic effects of BHB in microglia arises as a fundamental question. Here, we applied stable-isotope (^13^C) tracing and metabolomics to study the oxidative metabolism of BHB and the interplay between BHB and glucose metabolism in resting and LPS-activated microglia. We also assessed the effect of BHB on the inflammatory response to LPS by analysing changes in the expression of polarisation makers.

## 2. Results

### 2.1. Microglia Cells Oxidise β-Hydroxybutyrate in the TCA Cycle

**Figure 1:**
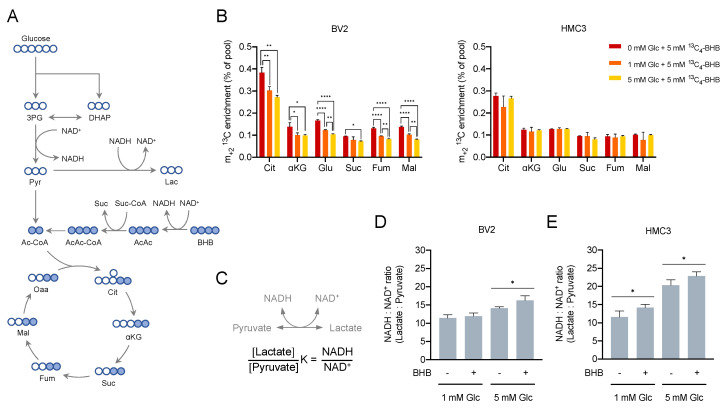
β-hydroxybutyrate (BHB) oxidation in murine (BV2) and human (HMC3) cells. (**A**) Schematic representation of ^13^C enrichment derived from ^13^C_4_-BHB. (**B**) m_+2_^13^C enrichment of TCA cycle intermediates (Cit, citrate; αKG, α-ketoglutarate; Glu, glutamate; Suc, succinate; Fum, fumarate; Mal, malate) in BV2 and HMC3 cells in culture conditions with either no added glucose or 1 or 5 mM of ^12^C_6_-glucose and 5 mM ^13^C_4_-BHB for 24 h. Bars represent mean ± SD of *n* = 3 biological replicates. Data were analysed by one-way ANOVA per metabolite followed by Tukey’s test. (**C**) Schematic representation of the cytosolic NADH:NAD^+^ ratio in equilibrium with Lactate:Pyruvate ratio. (**D**,**E**) Cytosolic NADH:NAD^+^ ratio estimated using the intracellular levels of lactate and pyruvate in BV2 (**D**) and HMC3 (**E**). Bars represent mean ± SD of *n* = 2–3 (-BHB) and *n* = 5–6 (+BHB) biological replicates. Data were analysed by two-way ANOVA followed by Sidak’s test (-BHB vs. +BHB within glucose class). Statistical significance is denoted as * *p* < 0.05, ** *p* < 0.01 and **** *p* < 0.0001.

To study the metabolism of BHB in microglia, the cell lines BV2 (mouse) and HMC3 (human) were chosen as microglia cell models. These cells conveniently recapitulate the most important aspects of the biology of the microglia and have been previously used in a number of studies [42,43,44]. The concentration of BHB rises in physiological conditions characterised by limited glucose availability [45]. Although oxidation of BHB has been long known to take place in neurons, astrocytes, and oligodendroglia [46,47,48,49], evidence of BHB oxidation in microglia is still lacking. We first sought to confirm whether microglia can oxidise BHB and to understand how glucose availability modulated BHB metabolism. To test these hypotheses, we performed a stable-isotope tracing experiment using ^13^C-labelled BHB. The use of ^13^C labelled substrates allows tracing of the fate of the carbons through the different metabolic pathways and the incorporation into downstream metabolites [50]. BHB is oxidised through the three-step ketone body oxidation pathway with the subsequent production of NADH and acetyl-CoA, which can be incorporated into the TCA cycle (Figure 1A). BV2 and HMC3 cultures were supplemented with 5 mM of uniformly ^13^C-labelled BHB (^13^C_4_-BHB) in culture conditions containing no added glucose, 1 or 5 mM of unlabelled glucose (^12^C_6_-glucose). A parallel analysis with uniformly ^13^C-labelled glucose (^13^C_6_-glucose) confirmed a significant decrease in the glycolytic flux in a glucose-limiting environment (Figure S1A,B). Our results showed that both BV2 and HMC3 can import and oxidise BHB, as indicated by the m_+2_^13^C enrichment in the TCA cycle intermediates citrate, α-ketoglutarate, glutamate, succinate, fumarate, and malate (Figure 1B). A complete list of mass isotopologues for all metabolites can be found in the Supplementary Results (Supplementary Table S1A,B). Our results also showed that glucose availability alters the fate of BHB in a different way in each cell line. In BV2 cells, the oxidation of BHB gradually increased as glucose concentration decreased, manifesting as a rise in m_+2_^13^C enrichment in all the TCA cycle intermediates (Figure 1B, left). HMC3 cells did not show the same pattern of response, and the oxidation of BHB remained constant irrespective of the glucose concentration, as indicated by a steady proportion of m_+2_^13^C enrichment across the TCA cycle intermediates (Figure 1B, right). Interestingly, in both BV2 and HMC3 cells, a detectable fraction of ^13^C_4_-BHB-derived carbons was transformed into lactate (Figure S2A,B) and pyruvate (Figure S2C,D). Specifically, we detected an increase in intracellular m_+2_^13^C_2_-lactate in a glucose-dependent fashion, suggesting the possibility of an alternative metabolic pathway of BHB that is enhanced under glucose-limiting conditions. It has been reported that microglia cells have a flexible metabolism and in conditions of glucose deprivation can rapidly shift to use glutamine to sustain mitochondrial metabolism and surveillance functions [17]. Thus, to test the possibility that BHB could rescue microglia proliferation in conditions of very low glucose, we cultured BV2 and HMC3 cells in 0.1 mM glucose supplemented with 5 or 10 mM unlabelled BHB. Our results indicate that BHB was not able to rescue proliferation in any of the cell lines (Figure S2E,F). Since both glucose and BHB fuel metabolic pathways involved in the production of NADH, we were interested in whether BHB could alter the bioenergetic metabolism and redox status by inducing changes in the NADH:NAD^+^ ratio. This ratio has been recently reported to control innate inflammatory responses through the transcriptional co-repressor CtBP [51]. The cytosolic NADH:NAD^+^ ratio can be indirectly estimated by measuring the ratio between the intracellular levels of lactate and pyruvate (Figure 1C) [51,52]. As expected, cells cultured in 5 mM glucose showed a higher NADH:NAD^+^ ratio than cells cultured in 1 mM glucose (Figure 1D,E). In BV2 cells, the addition of BHB increased the NADH:NAD^+^ ratio when cells were cultured in 5 mM glucose but not in 1 mM glucose (Figure 1D). In HMC3 cells, BHB addition raised the ratio both in 1 and 5 mM glucose conditions (Figure 1E). We also examined the effect of BHB supplementation on metabolite levels across multiple metabolic pathways in cells cultured in 1 and 5 mM glucose. The metabolome of BV2 cells was more responsive to BHB than HMC3. In 1 mM glucose, BV2 cells supplemented with BHB showed accumulation of lactate, glycine, and glutamate, whereas in 5 mM glucose, an accumulation of lactate and reduction in the concentration of glutamate was observed (Figure S3A). In HMC3 cells, BHB promoted accumulation of glutamate in cells cultured in low glucose (Figure S3B). Collectively, these data indicate that BHB is oxidised in the TCA cycle in microglia cells and promotes the production of NADH and upregulation of the cytosolic NADH:NAD^+^ ratio.

### 2.2. β-Hydroxybutyrate Modulates the LPS-Induced Glycolytic Response

**Figure 2:**
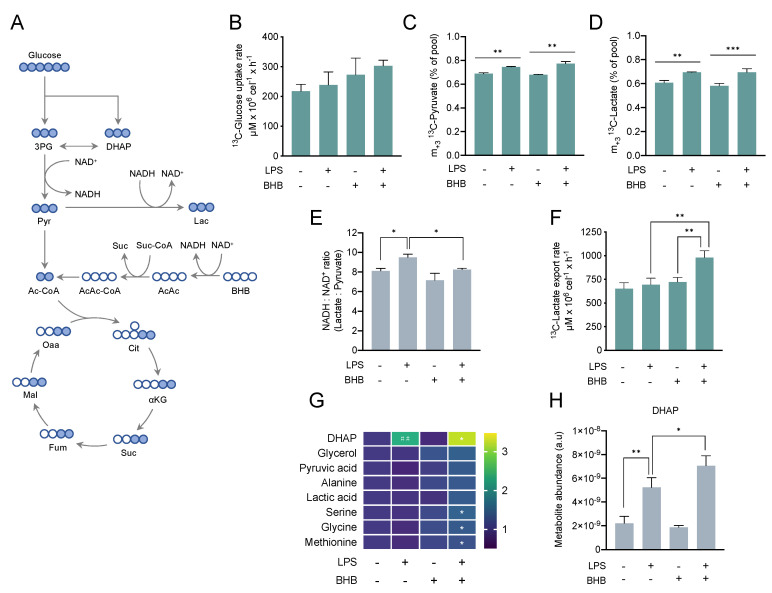
Glycolytic metabolism in response to lipopolysaccharide (LPS) and β-hydroxybutyrate (BHB) in BV2 cells. (**A**) Schematic representation of ^13^C enrichment derived from ^13^C_6_-glucose. In (**B**–**H**), cells were seeded and the next day treated with 5 mM ^12^C_4_-BHB for 24 h before the media was replaced by fresh media containing 5 mM ^13^C_6_-glucose and immediately treated with either 100 ng/mL of LPS, 5 mM of ^12^C_4_-BHB or both for 6 h. (**B**) ^13^C-glucose uptake rate in cells treated with 100 ng/mL LPS and/or 5 mM BHB for 6 h estimated by ^1^H-NMR. Two-way ANOVA (LPS n.s., BHB *p* = 0.027, LPS × BHB n.s.) followed by Tukey’s test (no statistical significance reached). (**C**) m_+3_^13^C enrichment in intracellular pyruvate. Data were analysed by two-way ANOVA (LPS *p* < 0.0001, BHB n.s., LPS × BHB *p* = 0.023) followed by Tukey’s test. (**D**) m_+3_^13^C enrichment in intracellular lactate. Data were analysed by two-way ANOVA (LPS *p* < 0.0001, BHB n.s., LPS × BHB n.s.) followed by Tukey’s test. (**E**) Cytosolic NADH:NAD+ ratio estimated using the intracellular levels of lactate and pyruvate. Data were analysed by two-way ANOVA (LPS *p* = 0.001, BHB *p* = 0.0021, LPS × BHB n.s.) followed by Tukey’s test. (**F**) ^13^C-lactate export rate calculated from ^13^C-lactate exported into media analysed by ^1^H-NMR. Data were analysed by two-way ANOVA (LPS *p* = 0.003, BHB *p* = 0.001, LPS × BHB *p* = 0.018) followed by Tukey’s test. (**G**) Metabolite levels normalised to untreated cells and expressed as fold-change as per colour-coded scale. Each data point represents mean of *n* = 3 biological replicates. Statistical significance was assessed by two-way ANOVA (DHAP: LPS *p* < 0.0001, BHB n.s., LPS × BHB *p* = 0.023; pyruvate, alanine, serine, glycine, and methionine: LPS n.s., BHB *p* < 0.05) followed by Tukey’s test (untreated vs. +LPS, untreated vs. +BHB, +LPS -BHB vs. +BHB +LPS). A complementary statistical analysis using Fisher’s LSD test followed by false discovery rate (FDR) correction to account for multiple comparisons both across treatment class and metabolite class can be found in Supplementary Table S3. (**H**) Bar chart representation of intracellular levels of DHAP. In (**B**–**E**,**H**), bars represent mean ± SD of *n* = 3 biological replicates. Statistical significance is denoted as * *p* < 0.05, ** *p* < 0.01, and *** *p* < 0.001 with exception of Figure 2G, where statistical significance between untreated and single treatments (untreated vs. +LPS, untreated vs. +BHB) is denoted as ## *p* < 0.01, and statistical significance between single treatment with LPS and double treatment (−BHB +LPS vs. +BHB +LPS) is denoted as * *p* < 0.05, ** *p* < 0.01, and *** *p* < 0.001.

The role of BHB in microglia and neuroinflammation has been previously studied in various disease models [26,36,38,53,54,55,56,57]. However, the metabolic effects of BHB on microglia activation and the underlying metabolic reprogramming remain unknown. Along these lines, an increasing body of evidence indicates that metabolic interference can modulate the microglia response to immune signals [6,7,51]. Thus, we sought to understand the impact of BHB on the metabolic reprogramming associated to LPS-activated microglia. Given that BV2 cells were metabolically more responsive to BHB addition and previous studies had optimised LPS stimulation and partially characterised the metabolic response of this cell line, BV2 was chosen as a model to explore the effects of BHB in LPS-induced metabolic reprogramming. BV2 cells were cultured in the presence of 5 mM ^13^C_6_-glucose and treated with either 5 mM ^12^C_4_-BHB, 100 ng/mL LPS, or both for 6 h. Successful activation was confirmed by the upregulation of the inflammatory marker *NOS2* (Figure S4A). To explore changes in the glycolytic metabolism, we measured the ^13^C enrichment and the relative abundance of key glycolytic intermediates (Figure 2A). While treatment with LPS did not change glucose uptake rate, we observed a trend of higher glucose uptake in cells treated with BHB alone or in combination with LPS, which did not reach statistical significance (Figure 2B). Addition of LPS, but not BHB, increased glycolytic flux based on the rise in intracellular m_+3_^13^C-pyruvate (Figure 2C) and m_+3_^13^C-lactate (Figure 2D). A complete list of mass isotopologues for all metabolites can be found in the Supplementary Results (Supplementary Table S2). Consistently, this increase in glycolytic flux when cells were treated with LPS was associated with a rise in the cytosolic NADH:NAD+ ratio (Figure 2E). Supplementation with BHB alone did not change the NADH:NAD+ ratio, but the combination of LPS and BHB reduced this compared to LPS alone. Interestingly, the effects of LPS and BHB on ^13^C-lactate export followed a different trend than the production of intracellular ^13^C-pyruvate and ^13^C-lactate. While separate treatments with LPS or BHB alone did not change ^13^C-lactate export rate, the combination treatment dramatically increased ^13^C-lactate export into the media (Figure 2F), suggesting that BHB could alter glucose-derived carbon fate and lactate metabolism and export.

We also studied changes in the relative abundance (pool size) of some glycolytic intermediates. We detected a large increase in the concentration of dihydroxyacetone phosphate (DHAP) (Figure 2G,H) in cells treated with LPS that increased higher when cells were simultaneously treated with LPS and BHB, suggesting a synergistic metabolic response to both agents. We also found a significant accumulation of serine, glycine, and methionine when cells were treated with LPS and BHB at the same time (Figure 2G). Collectively, these results demonstrate that LPS increases glycolytic flux and cytosolic NADH:NAD+ ratio and that BHB modulates the LPS-induced glycolytic phenotype by promoting lactate export and accumulation of glycolytic intermediates.

### 2.3. β-Hydroxybutyrate Promotes Mitochondrial Metabolism and Accumulation of TCA Cycle Intermediates

As previously shown in Figure 1B, microglia cells oxidise BHB in the TCA cycle. Over the last years, several TCA cycle intermediates have been reported to be involved in the signalling and regulation of immunity [58], but whether this metabolic regulation also occurs in microglia is still unclear.

**Figure 3:**
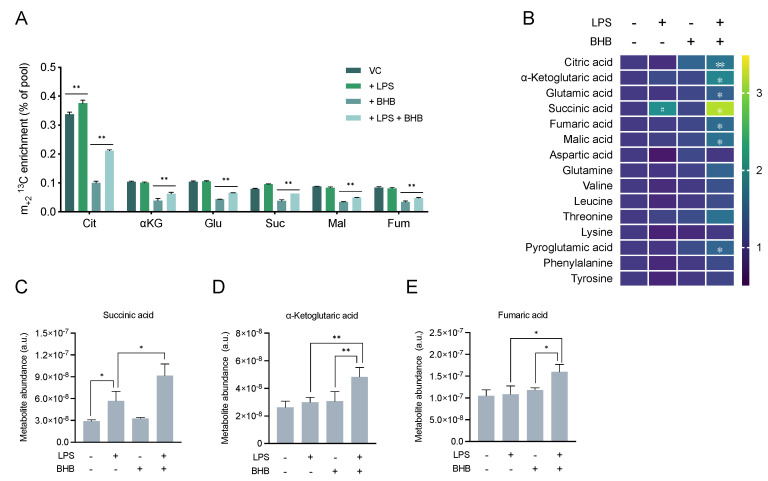
TCA cycle metabolism in response to lipopolysaccharide (LPS) and β-hydroxybutyrate (BHB) in BV2 cells. Experiment was performed as described in Figure 2. (**A**) m_+2_^13^C enrichment of TCA cycle intermediates (Cit, citrate; αKG, α-ketoglutarate; Glu, glutamate; Suc, succinate; Mal, malate; Fum, fumarate). Bars represent mean ± SD of *n* = 3 biological replicates. (**B**) Metabolite levels normalised to untreated cells and expressed as fold-change as per colour-coded scale. Each data point represents mean of *n* = 3 biological replicates. Statistical significance was assessed by two-way ANOVA (succinate: LPS *p* < 0.0001, BHB *p* = 0.011, LPS × BHB *p* = 0.029; α-ketoglutarate, fumarate, malate, aspartate: LPS *p* < 0.05; citrate, α-ketoglutarate, fumarate, malate, aspartate, and pyroglutamate: BHB *p* < 0.05) followed by Tukey’s test (untreated vs. +LPS, untreated vs. +BHB, -BHB +LPS vs. +BHB +LPS). A complementary statistical analysis using Fisher’s LSD test followed by false discovery rate (FDR) correction to account for multiple comparisons both across treatment class and metabolite class can be found in Supplementary Table S3. (**C**–**E**) Bar chart representation of the abundance of metabolites with known immunomodulatory activity. Bars represent mean ± SD of *n* = 3 biological replicates. Statistical significance is denoted as * *p* < 0.05, and ** *p* < 0.01, with exception of Figure 3B, where statistical significance between untreated and single treatments (untreated vs. +LPS, untreated vs. +BHB) is denoted as # *p* < 0.05, and statistical significance between single treatment with LPS and double treatment (−BHB + LPS vs. +BHB +LPS) is denoted as * *p* < 0.05 and ** *p* < 0.01.

To understand the impact of BHB in TCA cycle metabolism associated with LPS stimulation, we measured the ^13^C enrichment and relative abundance of the TCA cycle intermediates in BV2 cells in the same experimental conditions as in Figure 2. Treatment with LPS alone increased the flux of ^13^C-glucose-derived carbons into the mitochondria based on the increase in m_+2_^13^C-citrate (Figure 3A). Consistent with the results shown in Figure 1B, addition of BHB alone decreased the ^13^C enrichment in all TCA cycle intermediates compared to cells cultured in the absence of BHB due to dilution of the ^13^C labelling. Intriguingly, addition of LPS together with BHB largely increased the fraction of m_+2_^13^C-citrate and the other TCA cycle intermediates ^13^C-α-ketoglutarate, ^13^C-glutamate, ^13^C-succinate, ^13^C-malate, and ^13^C-fumarate compared to BHB alone (Figure 3A).

We also studied changes in the relative abundance of the TCA cycle intermediates and amino acids. Standalone treatment with LPS, but not BHB, substantially increased the level of succinate compared to untreated cells (Figure 3B,C), as previously reported in macrophages [59,60,61]. Strikingly, succinate levels were further increased in cells treated with LPS and BHB, suggesting a synergistic effect of combining the two exposures on the levels of this metabolite. Treatment with BHB alone did not significantly change the levels of any of the metabolites examined, but treatment with LPS and BHB together also increased the levels of the immunometabolites fumarate (Figure 3D) and α-ketoglutarate (Figure 3E). In addition, accumulation of citrate, glutamate, malate, and pyroglutamate was detected when cells were treated with LPS and BHB at the same time (Figure 3B). Taken together, our results indicate that BHB promotes mitochondrial metabolism and accumulation of TCA cycle immunometabolites in LPS-activated cells.

### 2.4. β-Hydroxybutyrate Enhances LPS-Induced Upregulation of the Pro-Inflammatory Marker NOS2

**Figure 4:**
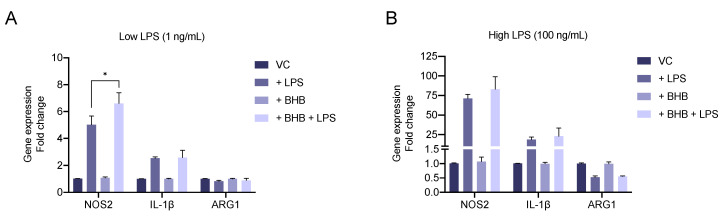
Effect of β-hydroxybutyrate (BHB) on BV2 polarisation after activation with lipopolysaccharide (LPS). Experiment was performed as indicated in Figure 2 using the unlabelled version for all metabolites. Gene expression of pro-inflammatory (*NOS2* and *IL-1β*) and anti-inflammatory (*ARG1*) polarisation markers in response to low (**A**) or high (**B**) stimulation with LPS in the presence or absence of 5 mM BHB after 6 h. Bars represent mean +/− SEM of *n* = 4 (*NOS2*), *n* = 2 (*IL-1β*) independent experiment and *n* = 2 (*ARG1*) biological replicates. Statistical significance was assessed by paired two-way ANOVA for individual genes followed by Sidak’s test (-BHB vs. +BHB within treatment class) ((**A**) *NOS2*: LPS *p* < 0.01, BHB *p* < 0.01, LPS × BHB *p* < 0.01; (**B**) *NOS2*: LPS *p* < 0.01, BHB n.s., LPS × BHB n.s.). Statistical significance is denoted as * *p* < 0.05.

In order to understand the impact of BHB on the activation and polarisation of microglia, BV2 cells were treated with a low (1 ng/mL) or high (100 ng/mL) dose of LPS, 5 mM BHB, or a combination of both, and expression of M1 (*NOS2* and *IL-1β*) and M2 (*ARG1*) polarisation markers was determined (Figure 4A,B). Stimulation with either a low or high concentration of LPS alone resulted in a strong upregulation of *NOS2* and *IL-1β* expression. Only cells treated with a high concentration of LPS significantly reduced the expression the *ARG1*. Addition of BHB alone did not affect the expression of any of the genes, but importantly, cells treated with a combination of a low dose of LPS and BHB showed enhanced upregulation of *NOS2* expression compared to cells treated with LPS alone.

## 3. Discussion

Herein, using stable-isotope tracing with ^13^C-BHB, we have shown that microglia cells can import and oxidise BHB in the TCA cycle with a subsequent increase in the cytosolic NADH:NAD+ ratio. Using ^13^C-glucose, we found that LPS upregulates glycolytic flux, increases NADH:NAD+ ratio, and promotes accumulation of DHAP. Addition of BHB enhanced LPS-induced accumulation of DHAP and promoted glucose-derived lactate export. BHB also synergistically increased LPS-induced accumulation of succinate and other key immunometabolites, such as α-ketoglutarate and fumarate generated by the TCA cycle. Finally, BHB upregulated the expression of a key pro-inflammatory (M1 polarisation) marker gene, *NOS2*, in microglia cells activated with LPS.

BHB is the principal ketone body, together with acetoacetate and acetone, and is synthesised in the liver from the oxidation of fatty acids derived from adipose tissue or diet. The basal concentration of BHB in plasma in healthy subjects is relatively low, with reference values reported 0.04–0.08 mM and typically <0.5 mM [62], and increases in specific conditions, such as a fasting (5–6 mM) [63], ketogenic diet (1 mM) [64,65] or diabetic ketoacidosis (>10 mM) [62].

Glucose is the preferred substrate of the brain [66]. Unlike most other tissues, the brain cannot utilize fatty acids for energy when blood glucose levels become compromised. However, during periods of low availability, it can be supplemented with the oxidation of alternative substrates like the monocarboxylates pyruvate [67], lactate [68,69], acetate [70], and ketone bodies [71,72]. Most of the current knowledge about the utilisation of alternative nutrients by brain cells has been obtained in neurons and astrocytes or in whole-brain experiments either in vivo or using cortical slices. Very little is known about the utilisation of alternative nutrients in the microglia [18]. Our results clearly demonstrate that microglia cells BV2 and HMC3 can import and oxidise the ketone body BHB. BHB is actively transported into the brain by the monocarboxylate transporters (MCT)—members of the SLC16 family that are proton symporters [73]. Although 14 members of the family have been identified so far, only MCT1, MCT2, and MCT4 have been clearly shown to be expressed in the central nervous system [74]. Of these, MCT1 and MCT2 have been confirmed to be expressed in microglia [75], with MCT2 being the one with the highest affinity for BHB with an estimated K_m_ of 1.2 mM, compared to 10.1 mM of MCT1 [76,77]. The oxidation of BHB to acetyl-CoA occurs through a linear sequence of metabolic reactions catalysed by the enzymes β-hydroxybutyrate dehydrogenase (BDH1/2), succinyl-CoA:3:oxoacid-CoA transferase (SCOT), and acetyl-CoA acetyltransferase (ACAT1/2) with the subsequent production of one molecule of NADH and succinate and two molecules of acetyl-CoA. SCOT is encoded by the gene OXCT1 and is considered to be the rate-limiting step in ketone body oxidation [78]. Our findings demonstrate that microglia, such as neurons, astrocytes, and oligodendrocytes [46,47], possess the enzymatic activity to oxidise ketone bodies. We also found that in BV2 cells, but not HMC3, uptake and oxidation of BHB can increase when cells are cultured in low glucose conditions. The different response between cell lines is difficult to explain, but it may well be related to the origin of each cell line. Several studies have reported differences in the enzyme activities involved in ketone body oxidation associated to age, species, and brain region [76]. Little is known about the regulation of ketone body oxidation (ketolysis). At the cellular level, ketogenesis is controlled by a regulatory network involving AMPK, mTOR, and PPARα [79]. Regarding ketolysis, in hepatocellular carcinoma, SCOT has been reported to be upregulated by a mTORC2-AKT-SP1 signalling axis promoting ketone body oxidation to provide energy and sustain tumour progression in nutritionally deprived environments [80]. Whether any of these pathways are involved in the regulation of ketolysis in microglia and could promote oxidation of BHB in glucose-limiting conditions remains to be explored.

It is understood that the ketone body oxidation pathway operates in the mitochondria [76], although a cytosolic form of BDH, known as BDH2 or DHRS6, has been reported in humans [81]. Our results showed a rise in the cytosolic NADH:NAD+ ratio in both cell lines supplemented with BHB, supporting the idea of ketolytic NADH production in the cytosol by BDH2. Changes in the cytosolic NADH:NAD+ ratio could also be related to indirect changes in the glycolytic flux due to transporter competition between BHB and lactate, as both molecules can be transported by the same MCTs. Experiments using ^2^H-BHB and detection of m_+1_ in glycolytic metabolites may help to shed light into this question.

We observed that a significant proportion of BHB is converted into pyruvate and lactate. Specifically, we detected a glucose-dependent increase in the intracellular m_+2_^13^C-lactate when cells were cultured with ^13^C_4_-BHB. We speculate that this observation is consistent with the use of the methylglyoxal pathway. It has been reported that patient-derived neutrophils can oxidise acetoacetate to methylglyoxal resulting in the production of D-lactate [82]. Through this pathway, BHB-derived acetoacetate can first be non-enzymatically transformed into acetone and next into methylglyoxal before being converted into lactate via the glyoxylase pathway. Alternatively, methylglyoxal can also be converted into pyruvate, via the enzyme betaine aldehyde dehydrogenase and 2-oxoaldehyde dehydrogenase, and subsequently into lactate by lactate dehydrogenase [83,84]. However, it is unclear whether the enzyme that converts acetone into methylglyoxal (NADPH-dependent CYPE21) is expressed in any tissue other than liver [85], although the existence of a non-enzymatic transformation involving copper ions has also been reported [84]. The activation of the methylglyoxal pathway and production of this metabolite has been associated with the induction of a proinflammatory phenotype in macrophages [86]. In addition, activation of macrophages and microglia with a combination of LPS and IFN-γ reportedly leads to the production and secretion of methylglyoxal [87].

We found a clear upregulation of glycolytic flux in cells treated with LPS, based on increased levels of m_+3_^13^C-lactate and m_+3_^13^C pyruvate in activated BV2 cells. These findings are in line with previous findings reported in microglia, where stimulation with proinflammatory signals has been related to increased glycolysis [11,12,88,89]. One of the most important changes that we observed was the accumulation of DHAP after treatment with LPS. DHAP is produced from fructose-1,6-biphosphate (F1,6BP) through the enzyme aldolase together with glyceraldehyde-3-phosphate (GAP). These compounds are isomers that can be readily interconverted. The isomerization of these three-carbon phosphorylated sugars is catalysed by triose phosphate isomerase (TPI) in a rapid and reversible reaction. At equilibrium, 96% of the triose phosphate is DHAP [90]. However, the reaction proceeds readily from DHAP to GAP because the subsequent reactions of glycolysis remove this product. GAP can be transformed into 1,3-biphosphoglycerate (1,3-BPG) by the action of the enzyme glyceraldehyde-3-phosphate dehydrogenase (GAPDH) [90]. GAPDH has been reported to be inhibited by nitric oxide (NO), the concentration of which increases in immune cells stimulated with LPS by upregulation of the proinflammatory gene *NOS2* [91,92,93,94,95,96]. Therefore, we reason that inhibition of GAPDH by NO in response to stimulation with LPS could lead to accumulation of G3P and DHAP.

Another important observation was the increase in lactate export only in cells treated with a combination of LPS and BHB. LPS increased the glycolytic flux without increasing the export of lactate but raising the fraction of glucose-derived carbons into the mitochondria (increased m_+2_^13^C-citrate). Addition of BHB to LPS-activated cells raised the influx of glucose-derived carbons into the mitochondria compared to BHB alone to a greater extent than LPS alone compared to untreated cells. This was accompanied by a large increase in the citrate pool. Based on that, we hypothesise that increased glycolytic flux and BHB oxidation might converge in the accumulation of high levels of acetyl-CoA. Acetyl-CoA acts as an allosteric inhibitor of the enzyme pyruvate dehydrogenase (PDH) and consequently reduces the conversion of pyruvate into acetyl-CoA [97], providing an excess of pyruvate for the lactate dehydrogenase reaction that can be converted into lactate and exported.

We also observed a significant accumulation of succinate in cells treated with LPS that increased more by cotreatment with BHB. Succinate is one of the metabolites known to have a strong immunomodulatory activity [20]. Accumulation of succinate in LPS-stimulated macrophages and microglia has been reported before [59,88,98]. Succinate was shown to stabilize hypoxia-inducible factor 1α that resulted in transcription of a number of inflammatory genes and was essential for glycolytic reprogramming [59]. One of the most striking observations was that addition of BHB resulted in further accumulation of succinate in LPS-activated cells, suggesting inhibition of succinate oxidation by succinate dehydrogenase (SDH). The combination of BHB and LPS, but not separate treatments, also resulted in accumulation of the other TCA cycle intermediates citrate, α-ketoglutarate, glutamate, malate, and fumarate. We reason that increased glycolytic flux and BHB oxidation might converge in the accumulation of high levels of acetyl CoA, citrate, and aconitate. This would in turn fuel the synthesis of itaconate, an offshoot from the TCA cycle made from aconitate by IRG1/aconitate decarboxylase 1 (ACOD1) [99]. Itaconate is an anti-inflammatory metabolite that inhibits SDH and promotes accumulation of succinate [61]. SDH inhibition together with oversupply of acetyl-CoA into the TCA cycle could led to further accumulation succinate and other TCA cycle intermediates, including itaconate.

We also found that BHB enhanced upregulation of *NOS2* expression, but not *IL-1β* or *ARG1*, in LPS-activated cells, suggesting a potential proinflammatory role of BHB in our experimental system. However, several studies have reported an anti-inflammatory role for BHB in different models of inflammation. This effect is largely mediated by activation of the GPR109A receptor [55,56,100,101,102] or direct inhibition of the NLRP3 inflammasome [33,41]. On the contrary, it has also been reported that BHB can increase *NOS2* expression in untreated primary microglia [56] and calf hepatocytes [103]. In our results, the fact that *NOS2* upregulation was only observed when cells were activated with a low dose of LPS but not with a high dose suggests that this phenomenon may not be detectable in more complex experimental settings and could be easily precluded in cells exposed to high stimulation or multiple immunological signals. Nevertheless, we believe that more work is needed to fully understand this regulatory mechanism.

BV2 cells are immortalized microglia cells that recapitulate a large proportion of the phenotypic traits of primary and in vivo microglia. They have been widely used to study microglia biology [42,43,44], and observations have successfully been validated in primary and in vivo microglia studies [104,105,106]. However, some limitations in the use of these cell lines have been reported. Such reports are mostly related to the limited capacity of these cells to fully capture the extent of changes after stimulation with different agents compared to primary or freshly isolated microglia [107,108]. Therefore, our observations should ideally be validated in advanced microglia models, such as primary microglia, induced pluripotent stem cells (iPSC)-derived microglia and in vivo studies.

In conclusion, we provide novel data defining the fate of BHB in microglia cell lines and demonstrate how BHB exposure increases the levels of known immunomodulatory metabolites in these models. Future work should be aimed at understanding the significance of these phenomena on immunological response in primary cells and in vivo, including study of the consequences of ketogenic diet in patients with chronic diseases, such as glioma. In syngeneic mouse models of glioma, the ketogenic diet led to complete tumour eradication when combined with radiation, suggesting the involvement of the immune system in bringing about this effect [109].

## 4. Materials and Methods

### 4.1. Cell Culture

BV2 cells (RRID:CVCL_0182) were kindly gifted by Prof. Joseph Bertrand (Karolinska Institute, Stockholm, Sweden). HMC3 cells (RRID:CVCL_II76) were purchased from ATCC (Ref. CRL-3304, May 2019). Both cell lines were cultured in DMEM (A1443001, Gibco) supplemented with 10% FBS (FBS Good, PAN Biotech), 2 mM glutamine (Gibco), and 5 mM glucose (Gibco) in a humidified atmosphere at 37 °C and 5% CO_2_. Cell lines were routinely tested for mycoplasma contamination.

### 4.2. Proliferation Assay

Cell growth was assessed by Sulforhodamine B (SRB) colorimetric assay according to manufacturer’s instructions [110]. BV2 and HMC3 cells were seeded in full media in 96-well plates at a density of 1 × 10^3^ and 3 × 10^3^ cells/well, respectively, and the next day media was replaced by test media. Nutrient composition of test media is reported in the figure legends and was replenished every day to control for changes in extracellular nutrient compositions.

### 4.3. Gene Expression Assay

RNA was extracted using Trizol^®^ (Invitrogen, ThermoFisher Scientific, Waltham, MA, USA) following manufacturer’s instructions. Briefly, cells were treated with Trizol^®^, and homogenates were collected and extracted by addition of chloroform followed by centrifugation at 12,000× *g* for 15 min at 4 °C. The top aqueous phase was collected, and RNA was precipitated by addition of isopropanol and subsequent centrifugation at 12,000× *g* for 10 min at 4 °C. Next, the supernatant was removed by aspiration, and the RNA pellet was rinsed with 75% ethanol. After ethanol aspiration, RNA was resuspended in RNAse-free water, and the concentration was determined using Nanodrop^TM^ (Thermo Scientific, ThermoFisher Scientific, Waltham, MA, USA). Next, cDNA was synthesized using 1 μg of RNA and M-MLV reverse transcriptase (Invitrogen, ThermoFisher Scientific) and random hexamers (Roche) as per manufacturer’s indications. Quantification of gene expression was performed by real-time PCR (CFX96, Bio-Rad) using SYBR Green (Applied Biosystems) following manufacturer’s instructions. The following primers were used: *NOS2*, FW 5′-CCCCGCTACTACTCCATCAG-3′, RV 5′-CCACTGACACTTCGCACAAA-3′; *IL-1β*, FW 5′-ACTCATTGTGGCTGTGGAGA-3′, RV 5′-TTGTTCATCTCGGAGCCTGT-3′; *ARG1*, FW 5′-ACTTCTGGGACTTCTGCCTC-3′, RV 5′-CGTAGTTGCCTCGGTTGATG-3′; *HPRT*, FW 5′-ATGGCCTCCCATCTCCTTCAT-3′, RV 5′-CAGTCCCAGCGTCGTGATTAG-3′. Primer efficiencies were calculated from the slope of a calibration curve generated using a pool of samples and were as follows: NOS2, 1.42; ARG1, 1.57; IL-1β, 1.48; HPRT, 2.13. Gene expression was determined accordingly by ∆∆Ct method using *HPRT* gene as a reference gene.

### 4.4. Stable-Isotope (^13^C) Tracing and Metabolic Profiling

Specific experimental conditions are detailed in the figure legends. Cells were seeded in 6-well plates and media was supplemented with ^12^C_6_-glucose (Sigma-Aldrich, St. Louis, MO, USA), ^13^C_6_-glucose (Sigma-Aldrich), ^12^C_4_-β-hydroxybutyrate (Sigma-Aldrich), and/or ^13^C_4_-β-hydroxybutyrate (Sigma-Aldrich). Media samples were collected at the beginning and end of the experiment for ^1^H-NMR analyses of metabolites. Metabolite extraction at the end of the experiment and subsequent derivatization and analysis were performed as described previously [111,112]. Briefly, cells were washed with Ringer’s buffer and quenched with 100% methanol and polar metabolites were extracted with 3:2:1 water: chloroform: methanol solution. Extracts were separated in a top aqueous fraction (polar metabolites) and a bottom organic fraction (non-polar metabolites). The aqueous fraction was dried down using a vacuum concentrator (Genevac^TM^ Fisher Scientific, ThermoFisher Scientific, Waltham, MA, USA) and subsequently subjected to methoxyamination with methoxamine (MOX) reagent (ThermoFisher Scientific) and derivatisation with MTBSTFA + 1% TBDMS (ThermoFisher Scientific). GC-MS analysis was performed on an Agilent 7890 GC equipped with a 30-m DB-5MS capillary column with a 10-m Duraguard column connected to an Agilent 5975 MSD operating under electron impact ionization. Data were acquired under full scan mode, and AMDIS (Automatic Mass Spectral Deconvolution and Integration System) software was used with reference to the NIST (National Institutes of Standards and Technology) mass spectral library to identify metabolites [113]. A list of the metabolites detected, retention times, and *m*/*z* clusters can be found in the Supplementary Methods. For determination of ^13^C enrichment, correction for natural abundance of elemental isotopes and isotopologue peak integration was done using in-house MATLAB^®^ (Mathworks, Natick, MA, USA) scripts based on GAVIN [114], and the abundance of each mass isotopologue was normalised by the sum of all the mass isotopologue abundances equal to one. For relative quantification of metabolites, the sum of the ion abundances in the cluster was normalised by the abundance of the internal standard d_27_-myristic acid and cell number. Metabolite abundances in arbitrary units are presented as fold change relative to untreated controls as indicated in figure legends.

### 4.5. ^1^H Nuclear Magnetic Resonance (NMR) Spectroscopy

Media samples for determination of ^13^C-glucose and ^13^C-lactate concentration were collected at the beginning and end of the experiment and centrifuged at 150× *g* for 5 min. A volume of 550 μL was transferred to a clean microcentrifuge tube. Subsequently, 50 μL of the internal calibration standard 4-4-dimethyl-4-silapentane-1-sulfonic acid in deuterium oxide (12 mM) was added before tubes were vortexed and centrifuged at 20,000× *g* for 1 min. Samples were transferred into 5 mm diameter NMR economy sample tubes (Wilmad-LabGlass). High-resolution 1-dimensional ^1^H NMR spectroscopy was performed using the 14.1 T Bruker AVANCE 400 MHz spectrometer (Bruker BioSpin, Billerica, MA, USA) at 298 K. NMR spectra were acquired using a conventional ZGPR solvent pre-saturation method with a single radiofrequency pulse, a recycle delay (d1) of 4 s, spectral width of 6402.049 Hz, 32 free induction decays and 64,000 data points. Data were automatically Fourier-transformed before being processed in MATLAB^®^ software (Mathworks, Natick, MA, USA) using in-house scripts developed by J.T. Pearce, H.C. Keun, T.M.D. Ebbels, C.H. Lau and R. Cavill at Imperial College London (London, UK). Phase correction, baseline correction, and normalisation to the internal standard reference peak was automatically done before spectral peaks were identified with reference to the Human Metabolome Database. Concentration of ^13^C-glucose and ^13^C-lactate was estimated by integration of the regions 5.41–5.48 ppm and 1.455–1.51 ppm, respectively, of the NMR spectra and followed by normalisation to the internal calibration standard. Representative NMR spectra are shown in Supplementary Figure S5. The rate of metabolite uptake and release was determined by calculating the difference in metabolite concentration [X] in samples at the start [X]_s_ and end [X]_e_ of the experiment according to the expression ∆[X] = [X]_e_ − [X]_s_. For normalisation, cell numbers were obtained from parallel plates at the start and end of the experiment. Cells were trypsinized and absolute cell numbers determined using the Vi-CELL^TM^ XR Cell Viability Analyser (Beckman Coulter, Indianapolis, IN, USA) were used to estimate the area under the curve (AUC). Finally, metabolic rates were calculated following the expression ∆[X]/AUC.

### 4.6. Statistical Analysis

Data were analysed using PRISM 8 (GraphPad Software). One-way or two-way ANOVA, Student’s *t*-test, and Fisher’s LSD test followed by appropriate correction for multiple comparisons were performed, as indicated in the figure legends. For simplicity, only relevant statistical comparisons were indicated in the figures. Statistical significance (*p*-value, *p*) was denoted as n.s., not significant, # or * *p* < 0.05, ## or ** *p* < 0.01, ### or *** *p* < 0.001 and ##### or **** *p* < 0.0001.

## Supplementary Materials

The following are available online at https://www.mdpi.com/2218-1989/10/9/346/s1. Figure S1: Glycolytic flux in cells cultured in 1 and 5 mM glucose conditions, Figure S2: BHB-derived pyruvate and lactate and effects of BHB on proliferation in very low glucose conditions, Figure S3: Metabolic profiling of BV2 and HMC3 cells treated with BHB in 1 and 5 mM glucose, Figure S4: *NOS2* expression in BV2 cells treated with LPS and BHB. Figure S5: Representative NMR spectra. Table S1: Complete mass isotopologue distributions of ^13^C-labelled metabolites of BV2 (S1A) and HMC3 (S1B) cells cultured with ^13^C_4_-BHB in different concentrations of glucose, Table S2: Complete mass isotopologue distributions of ^13^C-labelled metabolites of BV2 cells cultured with ^13^C_6_-glucose and treated with LPS and/or BHB. Table S3: Statistical analysis of metabolic profiling data presented in Figure 2 and Figure 3 using Fisher’s LSD test followed by FDR correction.

## References

[1] Salter M.W., Stevens B. (2017). Microglia emerge as central players in brain disease. Nat. Med..

[2] Jackson L., Dumanli S., Johnson M.H., Fagan S.C., Ergul A. (2020). Microglia knockdown reduces inflammation and preserves cognition in diabetic animals after experimental stroke. J. Neuroinflamm..

[3] Hambardzumyan D., Gutmann D.H., Kettenmann H. (2016). The role of microglia and macrophages in glioma maintenance and progression. Nat. Neurosci..

[4] Ransohoff R.M. (2016). A polarizing question: Do M1 and M2 microglia exist?. Nat. Neurosci..

[5] Lauro C., Limatola C. (2020). Metabolic Reprograming of Microglia in the Regulation of the Innate Inflammatory Response. Front. Immunol..

[6] Ghosh S., Castillo E., Frias E.S., Swanson R.A. (2018). Bioenergetic regulation of microglia. Glia.

[7] Borst K., Schwabenland M., Prinz M. (2018). Microglia metabolism in health and disease. Neurochem. Int..

[8] Baik S.H., Kang S., Lee W., Choi H., Chung S., Kim J.-I., Mook-Jung I. (2019). A Breakdown in Metabolic Reprogramming Causes Microglia Dysfunction in Alzheimer’s Disease. Cell Metab..

[9] Seim G.L., Britt E.C., John S.V., Yeo F.J., Johnson A.R., Eisenstein R.S., Pagliarini D.J., Fan J. (2019). Two-stage metabolic remodelling in macrophages in response to lipopolysaccharide and interferon-γ stimulation. Nat. Metab..

[10] Pearce E.L., Pearce E.J. (2013). Metabolic pathways in immune cell activation and quiescence. Immunity.

[11] Geric I., Schoors S., Claes C., Gressens P., Verderio C., Verfaillie C.M., Veldhoven P.P.V., Carmeliet P., Baes M. (2019). Metabolic Reprogramming during Microglia Activation. Immunometabolism.

[12] Orihuela R., McPherson C.A., Harry G.J. (2015). Microglial M1/M2 polarization and metabolic states. Br. J. Pharmacol..

[13] Caputa G., Castoldi A., Pearce E.J. (2019). Metabolic adaptations of tissue-resident immune cells. Nat. Immunol..

[14] Qiu J., Villa M., Sanin D.E., Buck M.D., O’Sullivan D., Ching R., Matsushita M., Grzes K.M., Winkler F., Chang C.-H. (2019). Acetate Promotes T Cell Effector Function during Glucose Restriction. Cell Rep..

[15] Davies L.C., Rice C.M., Palmieri E.M., Taylor P.R., Kuhns D.B., McVicar D.W. (2017). Peritoneal tissue-resident macrophages are metabolically poised to engage microbes using tissue-niche fuels. Nat. Commun..

[16] Angela M., Endo Y., Asou H.K., Yamamoto T., Tumes D.J., Tokuyama H., Yokote K., Nakayama T. (2016). Fatty acid metabolic reprogramming via mTOR-mediated inductions of PPARγ directs early activation of T cells. Nat. Commun..

[17] Bernier L., York E., Kamyabi A., Choi H., Weilinger N., MacVicar B. (2020). Microglial metabolic flexibility supports immune surveillance of the brain parenchyma. Nat. Commun..

[18] Nagy A.M., Fekete R., Horvath G., Koncsos G., Kriston C., Sebestyen A., Giricz Z., Kornyei Z., Madarasz E., Tretter L. (2018). Versatility of microglial bioenergetic machinery under starving conditions. Biochim. Biophys. Acta.

[19] De Goede K.E., Harber K.J., den Bossche J.V. (2019). Let’s Enter the Wonderful World of Immunometabolites. Trends Endocrinol. Metab..

[20] Zasłona Z., O’Neill L.A.J. (2020). Cytokine-like Roles for Metabolites in Immunity. Mol. Cell.

[21] Choi I.Y., Piccio L., Childress P., Bollman B., Ghosh A., Brandhorst S., Suarez J., Michalsen A., Cross A.H., Morgan T.E. (2016). A Diet Mimicking Fasting Promotes Regeneration and Reduces Autoimmunity and Multiple Sclerosis Symptoms. Cell Rep..

[22] Goldberg E.L., Molony R.D., Kudo E., Sidorov S., Kong Y., Dixit V.D., Iwasaki A. (2019). Ketogenic diet activates protective γδ T cell responses against influenza virus infection. Sci. Immunol..

[23] Goldberg E.L., Shchukina I., Asher J.L., Sidorov S., Artyomov M.N., Dixit V.D. (2020). Ketogenesis activates metabolically protective γδ T cells in visceral adipose tissue. Nat. Metab..

[24] Longo V.D., Mattson M.P. (2014). Fasting: Molecular mechanisms and clinical applications. Cell Metab..

[25] Orillion A.R., Damayanti N.P., Shen L., Adelaiye-Ogala R., Affronti H.C., Elbanna M., Chintala S., Ciesielski M.J., Fontana L., Kao C. (2018). Dietary protein restriction reprograms tumor associated macrophages and enhances immunotherapy. Clin. Cancer Res..

[26] Wu Z., Isik M., Moroz N., Steinbaugh M.J., Zhang P., Blackwell T.K. (2019). Dietary Restriction Extends Lifespan through Metabolic Regulation of Innate Immunity. Cell Metab..

[27] Soldati L., Renzo L.D., Jirillo E., Ascierto P.A., Marincola F.M., Lorenzo A.D. (2018). The influence of diet on anti-cancer immune responsiveness. J. Transl. Med..

[28] Klement R.J. (2017). Beneficial effects of ketogenic diets for cancer patients: A realist review with focus on evidence and confirmation. Med. Oncol..

[29] Klement R.J., Brehm N., Sweeney R.A. (2020). Ketogenic diets in medical oncology: A systematic review with focus on clinical outcomes. Med. Oncol..

[30] Woolf E.C., Syed N., Scheck A.C. (2016). Tumor Metabolism, the Ketogenic Diet and β-Hydroxybutyrate: Novel Approaches to Adjuvant Brain Tumor Therapy. Front. Mol. Neurosci..

[31] Gasior M., Rogawski M.A., Hartman A.L. (2006). Neuroprotective and disease-modifying effects of the ketogenic diet. Behav. Pharmacol..

[32] Lutas A., Yellen G. (2013). The ketogenic diet: Metabolic influences on brain excitability and epilepsy. Trends Neurosci..

[33] Youm Y.-H., Nguyen K.Y., Grant R.W., Goldberg E.L., Bodogai M., Kim D., D’Agostino D., Planavsky N., Lupfer C., Kanneganti T.D. (2015). The ketone metabolite β-hydroxybutyrate blocks NLRP3 inflammasome-mediated inflammatory disease. Nat. Med..

[34] Torres J.A., Kruger S.L., Broderick C., Amarlkhagva T., Agrawal S., Dodam J.R., Mrug M., Lyons L.A., Weimbs T. (2019). Ketosis Ameliorates Renal Cyst Growth in Polycystic Kidney Disease. Cell Metab..

[35] Newman J.C., Verdin E. (2017). β-Hydroxybutyrate: A Signaling Metabolite. Annu. Rev. Nutr..

[36] Trotta M.C., Maisto R., Guida F., Boccella S., Luongo L., Balta C., D’Amico G., Herman H., Hermenean A., Bucolo C. (2019). The activation of retinal HCA2 receptors by systemic beta-hydroxybutyrate inhibits diabetic retinal damage through reduction of endoplasmic reticulum stress and the NLRP3 inflammasome. PLoS ONE.

[37] Han Y.-M., Bedarida T., Ding Y., Somba B.K., Lu Q., Wang Q., Song P., Zou M.-H. (2018). β-Hydroxybutyrate Prevents Vascular Senescence through hnRNP A1-Mediated Upregulation of Oct4. Mol. Cell.

[38] Huang C., Wang P., Xu X., Zhang Y., Gong Y., Hu W., Gao M., Wu Y., Ling Y., Zhao X. (2018). The ketone body metabolite β-hydroxybutyrate induces an antidepression-associated ramification of microglia via HDACs inhibition-triggered Akt-small RhoGTPase activation. Glia.

[39] Chen Y., Ouyang X., Hoque R., Garcia-Martinez I., Yousaf M.N., Tonack S., Offermanns S., Dubuquoy L., Louvet A., Mathurin P. (2018). β-Hydroxybutyrate protects from alcohol-induced liver injury via a Hcar2-cAMP dependent pathway. J. Hepatol..

[40] Taggart A.K.P., Kero J., Gan X., Cai T.-Q., Cheng K., Ippolito M., Ren N., Kaplan R., Wu K., Wu T.-J. (2005). (D)-beta-Hydroxybutyrate inhibits adipocyte lipolysis via the nicotinic acid receptor PUMA-G. J. Biol. Chem..

[41] Goldberg E.L., Asher J.L., Molony R.D., Shaw A.C., Zeiss C.J., Wang C., Morozova-Roche L.A., Herzog R.I., Iwasaki A., Dixit V.D. (2017). β-Hydroxybutyrate Deactivates Neutrophil NLRP3 Inflammasome to Relieve Gout Flares. Cell Rep..

[42] Stansley B., Post J., Hensley K. (2012). A comparative review of cell culture systems for the study of microglial biology in Alzheimer’s disease. J. Neuroinflamm..

[43] Russo C.D., Cappoli N., Coletta I., Mezzogori D., Paciello F., Pozzoli G., Navarra P., Battaglia A. (2018). The human microglial HMC3 cell line: Where do we stand? A systematic literature review. J. Neuroinflamm..

[44] Timmerman R., Burm S.M., Bajramovic J.J. (2018). An Overview of in vitro Methods to Study Microglia. Front. Cell. Neurosci..

[45] Cotter D.G., Schugar R.C., Crawford P.A. (2013). Ketone body metabolism and cardiovascular disease. Am. J. Physiol.-Heart Circ. Physiol..

[46] Edmond J., Robbins R.A., Bergstrom J.D., Cole R.A., de Vellis J. (1987). Capacity for substrate utilization in oxidative metabolism by neurons, astrocytes, and oligodendrocytes from developing brain in primary culture. J. Neurosci. Res..

[47] Chechik T., Roeder L.M., Tildon J.T., Poduslo S.E. (1987). Ketone body enzyme activities in purified neurons, astrocytes and oligodendroglia. Neurochem. Int..

[48] Pan J.W., de Graaf R.A., Petersen K.F., Shulman G.I., Hetherington H.P., Rothman D.L. (2002). [2,4-^13^C_2_]-beta-Hydroxybutyrate metabolism in human brain. J. Cereb. Blood Flow Metab..

[49] Lopes-Cardozo M., Larsson O.M., Schousboe A. (1986). Acetoacetate and Glucose as Lipid Precursors and Energy Substrates in Primary Cultures of Astrocytes and Neurons from Mouse Cerebral Cortex. J. Neurochem..

[50] Jang C., Chen L., Rabinowitz J.D. (2018). Metabolomics and Isotope Tracing. Cell.

[51] Shen Y., Kapfhamer D., Minnella A.M., Kim J.-E., Won S.J., Chen Y., Huang Y., Low L.H., Massa S.M., Swanson R.A. (2017). Bioenergetic state regulates innate inflammatory responses through the transcriptional co-repressor CtBP. Nat. Commun..

[52] Williamson D., Lund P., Krebs H. (1967). The redox state of free nicotinamide-adenine dinucleotide in the cytoplasm and mitochondria of rat liver. Biochem. J..

[53] Lim S., Chesser A.S., Grima J.C., Rappold P.M., Blum D., Przedborski S., Tieu K. (2011). D-β-Hydroxybutyrate Is Protective in Mouse Models of Huntington’s Disease. PLoS ONE.

[54] Kajitani N., Iwata M., Miura A., Tsunetomi K., Yamanashi T., Matsuo R., Nishiguchi T., Fukuda S., Nagata M., Shibushita M. (2020). Prefrontal cortex infusion of beta-hydroxybutyrate, an endogenous NLRP3 inflammasome inhibitor, produces antidepressant-like effects in a rodent model of depression. Neuropsychopharmacol. Rep..

[55] Rahman M., Muhammad S., Khan M.A., Chen H., Ridder D.A., Müller-Fielitz H., Pokorná B., Vollbrandt T., Stölting I., Nadrowitz R. (2014). The β-hydroxybutyrate receptor HCA2 activates a neuroprotective subset of macrophages. Nat. Commun..

[56] Fu S.-P., Wang J.-F., Xue W.-J., Liu H.-M., Liu B., Zeng Y.-L., Li S.-N., Huang B.-X., Lv Q.-K., Wang W. (2015). Anti-inflammatory effects of BHBA in both in vivo and in vitro Parkinson’s disease models are mediated by GPR109A-dependent mechanisms. J. Neuroinflamm..

[57] Deora V., Albornoz E.A., Zhu K., Woodruff T.M., Gordon R. (2017). The Ketone Body β-Hydroxybutyrate Does Not Inhibit Synuclein Mediated Inflammasome Activation in Microglia. J. Neuroimmune Pharmacol..

[58] Ryan D.G., Murphy M.P., Frezza C., Prag H.A., Chouchani E.T., O’Neill L.A., Mills E.L. (2019). Coupling Krebs cycle metabolites to signalling in immunity and cancer. Nat. Metab..

[59] Tannahill G.M., Curtis A.M., Adamik J., Palsson-McDermott E.M., McGettrick A.F., Goel G., Frezza C., Bernard N.J., Kelly B., Foley N.H. (2013). Succinate is an inflammatory signal that induces IL-1β through HIF-1α. Nature.

[60] Cordes T., Wallace M., Michelucci A., Divakaruni A.S., Sapcariu S.C., Sousa C., Koseki H., Cabrales P., Murphy A.N., Hiller K. (2016). Immunoresponsive Gene 1 and Itaconate Inhibit Succinate Dehydrogenase to Modulate Intracellular Succinate Levels. J. Biol. Chem..

[61] Lampropoulou V., Sergushichev A., Bambouskova M., Nair S., Vincent E.E., Loginicheva E., Cervantes-Barragan L., Ma X., Huang S.C.-C., Griss T. (2016). Itaconate Links Inhibition of Succinate Dehydrogenase with Macrophage Metabolic Remodeling and Regulation of Inflammation. Cell Metab..

[62] Laffel L. (1999). Ketone bodies: A review of physiology, pathophysiology and application of monitoring to diabetes. Diabetes Metab. Res. Rev..

[63] Owen O.E., Felig P., Morgan A.P., Wahren J., Cahill G.F. (1969). Liver and kidney metabolism during prolonged starvation. J. Clin. Investig..

[64] Cohen C.W., Fontaine K.R., Arend R.C., Gower B.A. (2019). A Ketogenic Diet Is Acceptable in Women with Ovarian and Endometrial Cancer and Has No Adverse Effects on Blood Lipids: A Randomized, Controlled Trial. Nutr. Cancer.

[65] Klement R.J., Sweeney R.A. (2016). Impact of a ketogenic diet intervention during radiotherapy on body composition: I. Initial clinical experience with six prospectively studied patients. BMC Res. Notes.

[66] Mergenthaler P., Lindauer U., Dienel G.A., Meisel A. (2013). Sugar for the brain: The role of glucose in physiological and pathological brain function. Trends Neurosci..

[67] Gonzalez S.V., Nguyen N.H.T., Rise F., Hassel B. (2005). Brain metabolism of exogenous pyruvate: Brain metabolism of pyruvate. J. Neurochem..

[68] Boumezbeur F., Petersen K.F., Cline G.W., Mason G.F., Behar K.L., Shulman G.I., Rothman D.L. (2010). The contribution of blood lactate to brain energy metabolism in humans measured by dynamic 13C nuclear magnetic resonance spectroscopy. J. Neurosci..

[69] Mächler P., Wyss M.T., Elsayed M., Stobart J., Gutierrez R., von Faber-Castell A., Kaelin V., Zuend M., Martín A.S., Romero-Gómez I. (2016). In Vivo Evidence for a Lactate Gradient from Astrocytes to Neurons. Cell Metab..

[70] Rae C., Fekete A.D., Kashem M.A., Nasrallah F.A., Bröer S. (2012). Metabolism, Compartmentation, Transport and Production of Acetate in the Cortical Brain Tissue Slice. Neurochem. Res..

[71] Pan J.W., Rothman T.L., Behar K.L., Stein D.T., Hetherington H.P. (2000). Human brain beta-hydroxybutyrate and lactate increase in fasting-induced ketosis. J. Cereb. Blood Flow Metab..

[72] Achanta L.B., Rowlands B.D., Thomas D.S., Housley G.D., Rae C.D. (2017). β-Hydroxybutyrate Boosts Mitochondrial and Neuronal Metabolism but is not Preferred Over Glucose Under Activated Conditions. Neurochem. Res..

[73] Halestrap A.P. (2013). The SLC16 gene family-structure, role and regulation in health and disease. Mol. Asp. Med..

[74] Pellerin L., Bergersen L.H., Halestrap A.P., Pierre K. (2005). Cellular and subcellular distribution of monocarboxylate transporters in cultured brain cells and in the adult brain. J. Neurosci. Res..

[75] Moreira T.J.T.P., Pierre K., Maekawa F., Repond C., Cebere A., Liljequist S., Pellerin L. (2009). Enhanced cerebral expression of MCT1 and MCT2 in a rat ischemia model occurs in activated microglial cells. J. Cereb. Blood Flow Metab..

[76] Achanta L.B., Rae C.D. (2016). β-Hydroxybutyrate in the Brain: One Molecule, Multiple Mechanisms. Neurochem. Res..

[77] Bröer S., Bröer A., Schneider H.-P., Stegen C., Halestrap A.P., Deitmer J.W. (1999). Characterization of the high-affinity monocarboxylate transporter MCT2 in Xenopus laevis oocytes. Biochem. J..

[78] Fukao T., Lopaschuk G.D., Mitchell G.A. (2004). Pathways and control of ketone body metabolism: On the fringe of lipid biochemistry. Prostaglandins Leukot Essent. Fat. Acids.

[79] Grabacka M., Pierzchalska M., Dean M., Reiss K. (2016). Regulation of Ketone Body Metabolism and the Role of PPARα. Int. J. Mol. Sci..

[80] Huang D., Li T., Wang L., Zhang L., Yan R., Li K., Xing S., Wu G., Hu L., Jia W. (2016). Hepatocellular carcinoma redirects to ketolysis for progression under nutrition deprivation stress. Cell Res..

[81] Guo K., Lukacik P., Papagrigoriou E., Meier M., Lee W.H., Adamski J., Oppermann U. (2005). Characterization of Human DHRS6, an Orphan Short Chain Dehydrogenase/Reductase Enzyme: A novel, cytosolic type 2 R-β-hydroxybutyrate dehydrogenase. J. Biol. Chem..

[82] Aleksandrovskii Y.A. (1992). Antithrombin III, C1 inhibitor, methylglyoxal, and polymorphonuclear leukocytes in the development of vascular complications in diabetes mellitus. Thromb. Res..

[83] Glew R.H. (2010). You can get there from here: Acetone, anionic ketones and even-carbon fatty acids can provide substrates for gluconeogenesis. Niger. J. Physiol. Sci. Off. Publ. Physiol. Soc. Niger..

[84] Jagt D.L.V., Hassebrook R.K., Hunsaker L.A., Brown W.M., Royer R.E. (2001). Metabolism of the 2-oxoaldehyde methylglyoxal by aldose reductase and by glyoxalase-I: Roles for glutathione in both enzymes and implications for diabetic complications. Chem. Biol. Interact..

[85] Casazza J.P., Felver M.E., Veech R.L. (1984). The metabolism of acetone in rat. J. Biol. Chem..

[86] Bhattacharyya N., Pal A., Patra S., Haldar A.K., Roy S., Ray M. (2008). Activation of macrophages and lymphocytes by methylglyoxal against tumor cells in the host. Int. Immunopharmacol..

[87] Dhananjayan K., Gunawardena D., Hearn N., Sonntag T., Moran C., Gyengesi E., Srikanth V., Münch G. (2017). Activation of Macrophages and Microglia by Interferon-γ and Lipopolysaccharide Increases Methylglyoxal Production: A New Mechanism in the Development of Vascular Complications and Cognitive Decline in Type 2 Diabetes Mellitus?. J. Alzheimers Dis..

[88] Nair S., Sobotka K.S., Joshi P., Gressens P., Fleiss B., Thornton C., Mallard C., Hagberg H. (2019). Lipopolysaccharide-induced alteration of mitochondrial morphology induces a metabolic shift in microglia modulating the inflammatory response in vitro and in vivo. Glia.

[89] Gimeno-Bayón J., López-López A., Rodríguez M.J., Mahy N. (2014). Glucose pathways adaptation supports acquisition of activated microglia phenotype. J. Neurosci. Res..

[90] Berg J., Tymoczko J., Stryer L. (2002). Section 16.1, Glycolysis Is an Energy-Conversion Pathway in Many Organisms. Biochemistry.

[91] Hara M.R., Agrawal N., Kim S.F., Cascio M.B., Fujimuro M., Ozeki Y., Takahashi M., Cheah J.H., Tankou S.K., Hester L.D. (2005). S-nitrosylated GAPDH initiates apoptotic cell death by nuclear translocation following Siah1 binding. Nat. Cell Biol..

[92] Mohr S., Stamler J.S., Brüne B. (1996). Posttranslational Modification of Glyceraldehyde-3-phosphate Dehydrogenase by S -Nitrosylation and Subsequent NADH Attachment. J. Biol. Chem..

[93] Souza J.M., Radi R. (1998). Glyceraldehyde-3-Phosphate Dehydrogenase Inactivation by Peroxynitrite. Arch. Biochem. Biophys..

[94] Dimmeler S., Lottspeich F., Brüne B. (1992). Nitric oxide causes ADP-ribosylation and inhibition of glyceraldehyde-3-phosphate dehydrogenase. J. Biol. Chem..

[95] Dimmeler S., Ankarcrona M., Nicotera P., Brüne B. (1993). Exogenous nitric oxide (NO) generation or IL-1 beta-induced intracellular NO production stimulates inhibitory auto-ADP-ribosylation of glyceraldehyde-3-phosphate dehydrogenase in RINm5F cells. J. Immunol..

[96] Y Vedia L.M., McDonald B., Reep B., Brüne B., Silvio M.D., Billiar T.R., Lapetina E.G. (1992). Nitric oxide-induced S-nitrosylation of glyceraldehyde-3-phosphate dehydrogenase inhibits enzymatic activity and increases endogenous ADP-ribosylation. J. Biol. Chem..

[97] Berg J., Tymoczko J., Stryer L. (2002). Section 17.2, Entry to the Citric Acid Cycle and Metabolism through It Are Controlled. Biochemistry.

[98] Mills E.L., Kelly B., Logan A., Costa A.S.H., Varma M., Bryant C.E., Tourlomousis P., Däbritz J.H.M., Gottlieb E., Latorre I. (2016). Succinate Dehydrogenase Supports Metabolic Repurposing of Mitochondria to Drive Inflammatory Macrophages. Cell.

[99] Michelucci A., Heurtaux T., Grandbarbe L., Morga E., Heuschling P. (2009). Characterization of the microglial phenotype under specific pro-inflammatory and anti-inflammatory conditions: Effects of oligomeric and fibrillar amyloid-beta. J. Neuroimmunol..

[100] Graff E.C., Fang H., Wanders D., Judd R.L. (2016). Anti-inflammatory effects of the hydroxycarboxylic acid receptor 2. Metab. Clin. Exp..

[101] Offermanns S., Schwaninger M. (2015). Nutritional or pharmacological activation of HCA(2) ameliorates neuroinflammation. Trends Mol. Med..

[102] Zandi-Nejad K., Takakura A., Jurewicz M., Chandraker A.K., Offermanns S., Mount D., Abdi R. (2013). The role of HCA2 (GPR109A) in regulating macrophage function. FASEB J..

[103] Shi X., Li X., Li D., Li Y., Song Y., Deng Q., Wang J., Zhang Y., Ding H., Yin L. (2014). β-Hydroxybutyrate activates the NF-κB signaling pathway to promote the expression of pro-inflammatory factors in calf hepatocytes. Cell. Physiol. Biochem..

[104] Henn A., Lund S., Hedtjärn M., Schrattenholz A., Pörzgen P., Leist M. (2009). The suitability of BV2 cells as alternative model system for primary microglia cultures or for animal experiments examining brain inflammation. Altex.

[105] Carrillo-Jimenez A., Deniz Ö., Niklison-Chirou M.V., Ruiz R., Bezerra-Salomão K., Stratoulias V., Amouroux R., Yip P.K., Vilalta A., Cheray M. (2019). TET2 Regulates the Neuroinflammatory Response in Microglia. Cell Rep..

[106] Burguillos M.A., Deierborg T., Kavanagh E., Persson A., Hajji N., Garcia-Quintanilla A., Cano J., Brundin P., Englund E., Venero J.L. (2011). Caspase signalling controls microglia activation and neurotoxicity. Nature.

[107] Das A., Kim S.H., Arifuzzaman S., Yoon T., Chai J.C., Lee Y.S., Park K.S., Jung K.H., Chai Y.G. (2016). Transcriptome sequencing reveals that LPS-triggered transcriptional responses in established microglia BV2 cell lines are poorly representative of primary microglia. J. Neuroinflamm..

[108] Butovsky O., Jedrychowski M.P., Moore C.S., Cialic R., Lanser A.J., Gabriely G., Koeglsperger T., Dake B., Wu P.M., Doykan C.E. (2013). Identification of a unique TGF-β-dependent molecular and functional signature in microglia. Nat. Neurosci..

[109] Abdelwahab M.G., Fenton K.E., Preul M.C., Rho J.M., Lynch A., Stafford P., Scheck A.C. (2012). The ketogenic diet is an effective adjuvant to radiation therapy for the treatment of malignant glioma. PLoS ONE.

[110] Vichai V., Kirtikara K. (2006). Sulforhodamine B colorimetric assay for cytotoxicity screening. Nat. Protoc..

[111] Barnes E.M.E., Xu Y., Benito A., Herendi L., Siskos A.P., Aboagye E.O., Nijhuis A., Keun H.C. (2020). Lactic acidosis induces resistance to the pan-Akt inhibitor uprosertib in colon cancer cells. Br. J. Cancer.

[112] Lau C.-H.E., Tredwell G.D., Ellis J.K., Lam E.W.-F., Keun H.C. (2017). Metabolomic characterisation of the effects of oncogenic PIK3CA transformation in a breast epithelial cell line. Sci. Rep..

[113] Siderius D. NIST Standard Reference Simulation Website. https://www.nist.gov/programs-projects/nist-standard-reference-simulation-website.

[114] Behrends V., Tredwell G.D., Bundy J.G. (2011). A software complement to AMDIS for processing GC-MS metabolomic data. Anal. Biochem..

