# Supplementary material for: β-Hydroxybutyrate Oxidation Promotes the Accumulation of Immunometabolites in Activated Microglia Cells
Source: Metabolites. 2020 Aug 26;10(9):346. doi: 10.3390/metabo10090346 (PMC7570092; doi:10.3390/metabo10090346)
Supplement: Supplementary file 1 [file metabolites-10-00346-s001.zip › metabolites-872659-supplementary-proofed/Supplementary Methods.pdf]

β-hydroxybutyrate oxidation promotes the accumulation of immunometabolites in activated microglia cells

Supplementary Methods

Metabolite	RT	m/z range	Formula
Pyruvic acid	10.39	174-177	$C_3H_4O_3$
Lactic acid	13.30	261-264	$C_3H_6O_3$
Alanine	13.88	260-263	$C_3H_7NO_2$
Glycine	14.20	246-248	$C_2H_5NO_2$
Valine	15.24	288-293	$C_5H_{11}NO_2$
Leucine	16.09	302-308	$C_6H_{13}NO_2$
Isoleucine	16.10	302-308	$C_6H_{13}NO_2$
Succinic acid	16.49	289-293	$C_4H_6O_4$
Fumaric acid	16.84	287-291	$C_4H_4O_4$
Glycerol	17.60	377-380	$C_3H_8O_3$
Pyroglutamic acid	18.42	300-305	$C_5H_7NO_3$
Methionine	18.56	320-325	$C_5H_{11}NO_2S$
Serine	18.71	390-393	$C_3H_7NO_3$
α-Ketoglutaric acid	18.92	346-351	$C_5H_6O_5$
Threonine	19.00	404-408	$C_4H_9NO_3$
Phenylalanine	19.73	336-345	$C_9H_{11}NO_2$
Malic acid	19.88	419-423	$C_4H_6O_5$
Aspartic acid	20.28	418-422	$C_4H_7NO_4$
Glutamic acid	21.34	432-437	$C_5H_9NO_4$
Dihydroxyacetone phosphate	22.05	484-487	$C_3H_7O_6P$
Lysine	22.29	431-437	$C_6H_{14}N_2O_2$
Glutamine	22.65	431-436	$C_5H_{10}N_2O_3$
Glycerol-3-phosphate	23.38	571-574	$C_3H_9O_6P$
Citric acid	24.05	591-597	$C_6H_8O_7$
Tyrosine	24.33	466-475	$C_9H_{11}NO_3$

Table of metabolites detected by GC-MS. Retention times (RT) may vary depending on chromatographic conditions.
