# Supplementary material for: β-Hydroxybutyrate Oxidation Promotes the Accumulation of Immunometabolites in Activated Microglia Cells
Source: Metabolites. 2020 Aug 26;10(9):346. doi: 10.3390/metabo10090346 (PMC7570092; doi:10.3390/metabo10090346)
Supplement: Supplementary file 1 [file metabolites-10-00346-s001.zip › metabolites-872659-supplementary-proofed/Supplementary Results - figures.pdf]

β-hydroxybutyrate oxidation promotes the accumulation of immunometabolites in activated microglia cells

Supplementary Results

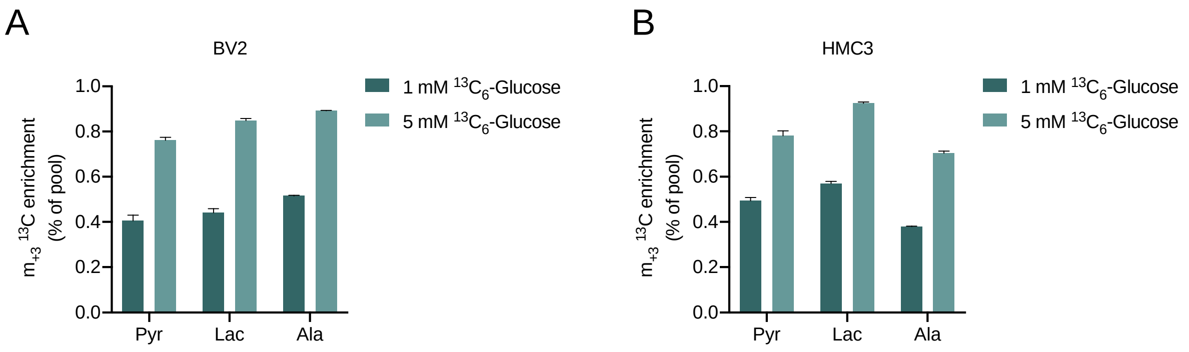

Figure S1. Glycolytic flux in cells cultured in 1 and 5 mM glucose conditions.
^{13}C enrichment of glycolytic metabolites (Pyr, pyruvate; Lac, lactate; Ala, alanine) in BV2 (A) and HMC3 (B) cells cultured in 1 or 5 mM $^{13}C_6$-glucose for 24h. Bars represent mean ± SD of n=3 biological replicates.

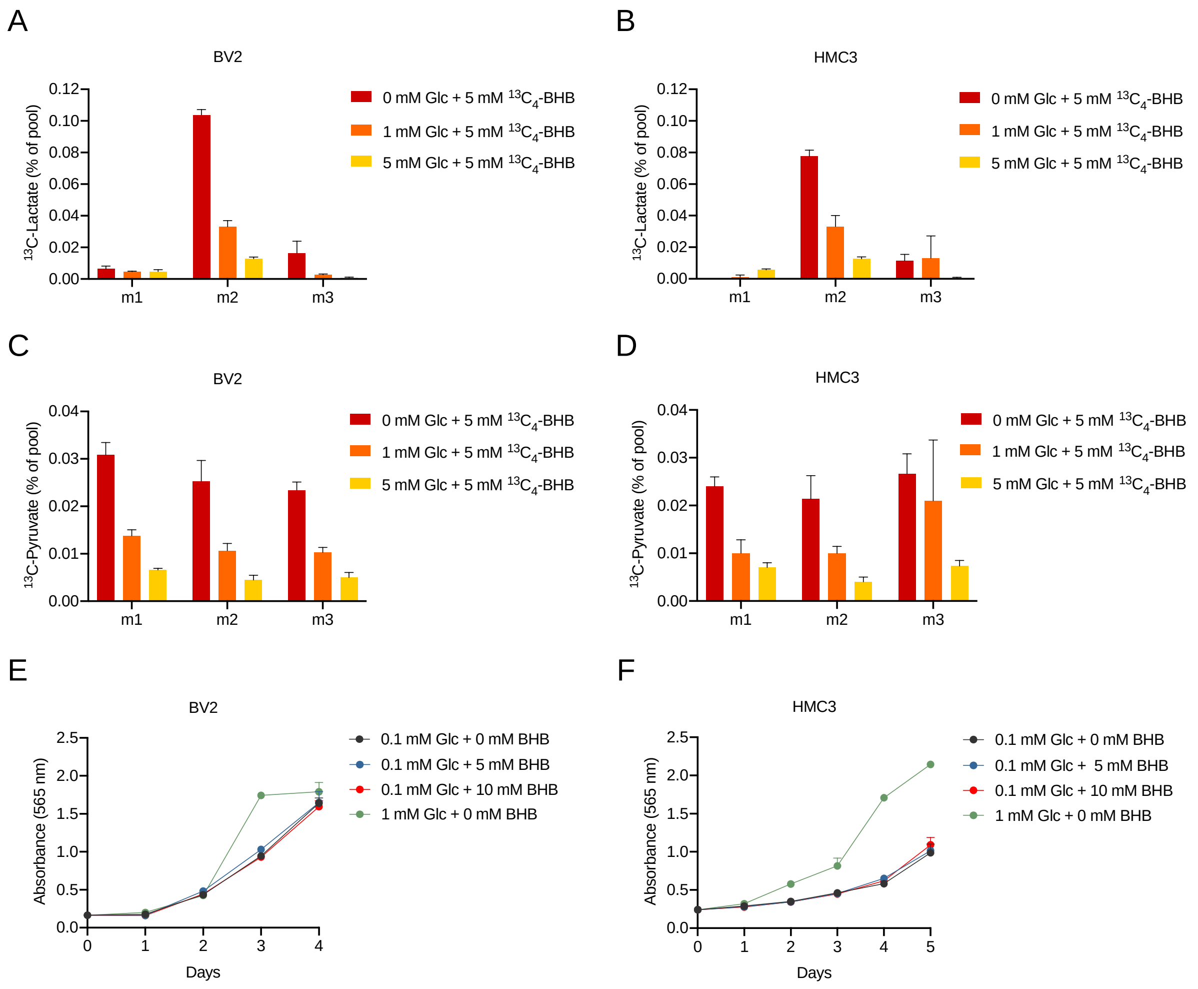

Figure S2. BHB-derived pyruvate and lactate and effects of BHB on proliferation in very low glucose conditions.

(A-D) ^{13}C enrichment of intracellular lactate and pyruvate in BV2 and HMC3 cells cultured with 5 mM $^{13}C_4$-BHB under conditions containing no glucose added, 1 mM or 5 mM unlabelled glucose for 24h. In all cases bars represent mean ± SD of n=3 biological replicates (with exception of HMC3 1 mM glucose where n=2).

(E-F) Proliferation of BV2 (E) and HMC3 (F) cells in 0.1 mM glucose supplemented with 5 or 10 mM BHB in media containing 10% dialysed FBS. Proliferation in 1 mM glucose is shown as a control. Each data point represent mean ± SD of n=3 biological replicates.

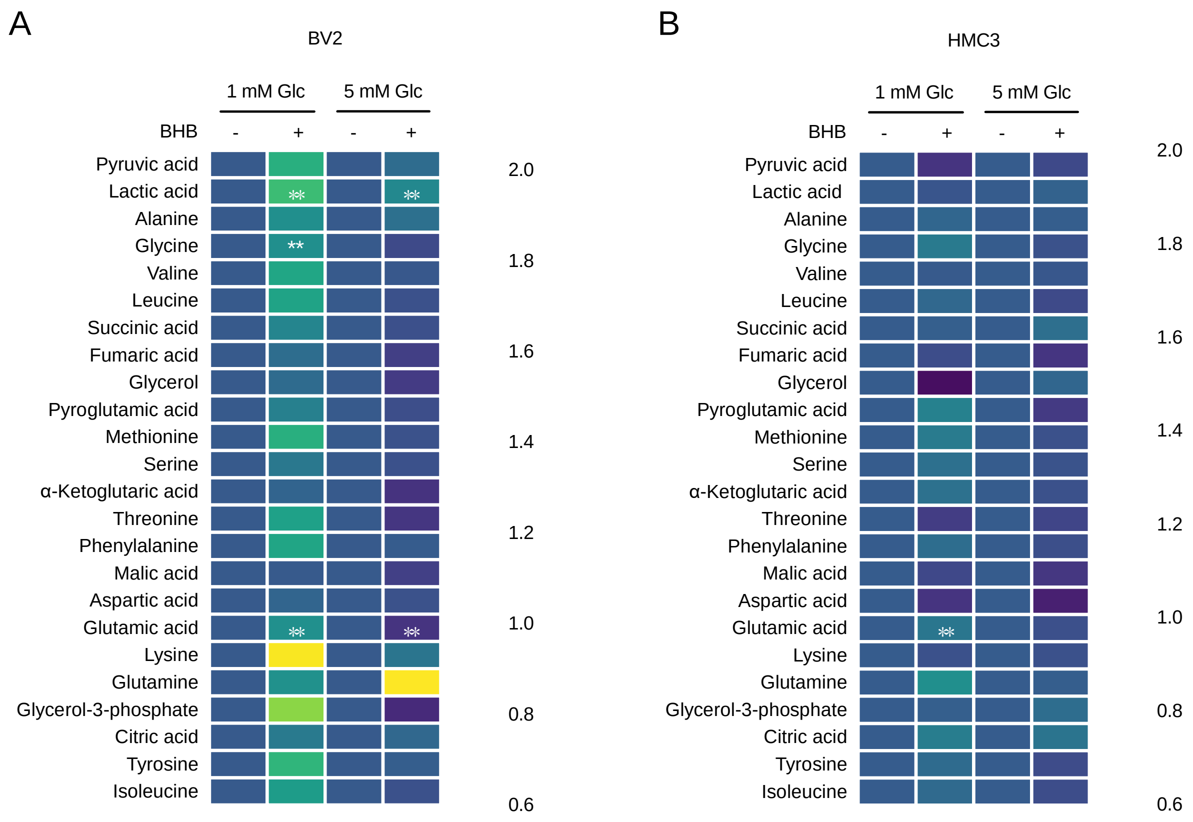

Figure S3. Metabolic profiling of BV2 and HMC3 cells treated with BHB in 1 and 5 mM glucose.

Metabolite levels in BV2 (A) and HMC3 (B) cells supplemented with 5 mM BHB in 1 mM and 5 mM glucose for 24h. Metabolite levels were normalised by untreated control for each glucose condition and expressed as fold-change as per colour-coded scale. Each data point represents mean of n=2-3 (-BHB) and n=5-6 (+BHB) biological replicates. Statistical differences were assessed by unpaired t-test for each metabolite (-BHB vs. +BHB within glucose class) followed by Hold-Sidak correction for multiple comparisons.

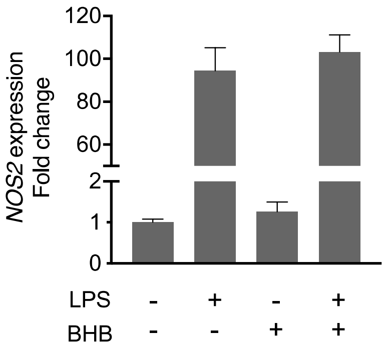

Figure S4. *NOS2* expression in BV2 cells treated with LPS and BHB.

Expression of *NOS2* in BV2 cells treated with 100 ng/mL LPS and/or 5 mM BHB for 6h.

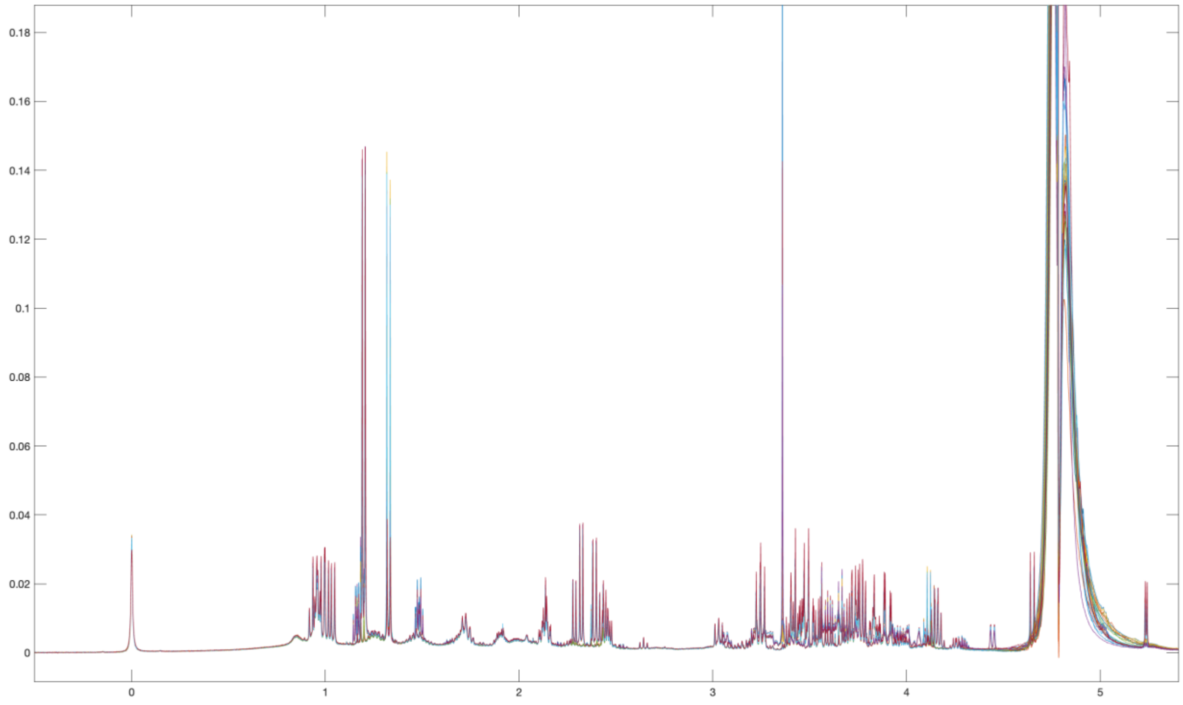

Figure S5. Representative NMR spectra

NMR spectra of media samples collected at the beginning and at the end of a $^{13}C_6$-glucose labelling experiment where cells were treated with BHB (5 mM) and/or LPS (100 ng/mL). Samples from cultures obtained in the same conditions using $^{12}C_6$-glucose are also included.
